# Ancestral feeding state of ruminants reconsidered: earliest grazing adaptation claims a mixed condition for Cervidae

**DOI:** 10.1186/1471-2148-8-13

**Published:** 2008-01-18

**Authors:** Daniel DeMiguel, Mikael Fortelius, Beatriz Azanza, Jorge Morales

**Affiliations:** 1 Departamento de Paleobiología, Museo Nacional de Ciencias Naturales, Consejo Superior de Investigaciones Científicas, José Gutiérrez Abascal 2, 28006 Madrid, Spain; 2 Departamento de Ciencias de la Tierra, Facultad de Ciencias, 50009, Universidad de Zaragoza, Spain; 3 Department of Geology, Division of Geology and Paleontology, PO Box 11 FIN-00014, University of Helsinki, Finland

## Abstract

**Background:**

Specialised leaf-eating is almost universally regarded as the ancestral state of all ruminants, yet little evidence can be cited in support of this assumption, apart from the fact that all early ruminants had low crowned cheek teeth. Instead, recent years have seen the emergence evidence contradicting the conventional view that low tooth crowns always indicate leaf-eating and high tooth crowns grass-eating.

**Results:**

Here we report the results of two independent palaeodietary reconstructions for one of the earliest deer, *Procervulus ginsburgi *from the Early Miocene of Spain, suggesting that despite having lower tooth crowns than any living ruminant, this species included a significant proportion of grass in its diet.

**Conclusion:**

The phylogenetic distribution of feeding styles strongly supports that leaf-grass mixed feeding was the original feeding style of deer, and that later dietary specialization on leaves or grass occurred independently in several lineages. Evidence for other ruminant clades suggests that facultative mixed feeding may in fact have been the primitive dietary state of the Ruminantia, which would have been morphologically expressed only under specific environmental factors.

## Background

The evolutionary strategies and feeding preferences in extinct ruminants have figured importantly in evolutionary ecology [1-9], and although a more complex view of the dietary adaptations and the evolutionary transitions of the feeding styles have recently begun to emerge [4,5], little reliable dietary information exists for the taxa involved in the Early Miocene ruminant radiation [10-14]. Because increased molar crown height (hypsodonty) has long been considered an evolutionary response to grass eating (grazing), the presence of low-crowned molars (brachydonty) is often thought to imply a lack of grass in the diet, and thus a leaf-eating (browsing) feeding style [3]. This notion has led to browsing being widely recognized to be the ancestral feeding state of ruminants and the starting point of the evolutionary diversification towards mixed feeders and grazers. Thus Early and Middle Miocene deer have until now been universally considered as tropical and subtropical forest dwelling browsers [11,15], despite the fact that many modern cervids exhibit strategies typical of mixed feeders, and even of grazers [5], while being significantly more brachydont than most other grass-eating ungulates.

The oldest ruminants bearing antler-like appendages are first recorded in the Early Miocene (MN3) of Europe. Among them, *Procervulus *and *Acteocemas *have been considered related to true antlered deer, but the only teeth of *Procervulus *are known, and so the investigated here. The central fold on the protocone of upper molars, as well as the dichotomous branching pattern and the furrowed surface of the protoantler of *Procervulus*, place it close to cervids, either as the sister-group [16] or the most basal membership [17,18] of the family Cervidae. Some specimens might be assigned to the genus *Procervulus *at this early time in the Spanish Neogene, and *Procervulus ginsburgi *[19]from the MN4 locality of Artesilla (Calatayud-Daroca basin, Aragonian type-area) is the oldest species represented by sufficient material to allow reconstruction of its palaeodiet. *P. ginsburgi *is therefore a key to understanding the most likely initial hypsodonty, feeding regime and habitat use in the first deer. *Dicrocerus elegans *from Sansan (France), MN6 and approximately 3 Ma younger than *P. ginsburgi *[20], is another early known deer for which dietary adaptations have been also studied [13].

Therefore, it is provided here a research for testing the hypothesis which concerns the ancestral leaf-eating state of the Cervidae. If this hypothesis is correct, it is to be expected that *P. ginsburgi*, one of the oldest and most brachydont known deer, reflects a dietary strategy equaling that of specialised browsers. To analyze the coevolutionary relationship between molar crown height and feeding behaviour, we undertook a study of hypsodonty [3,21,22] coupled with a combined analysis of microwear [6,8,10,23,24] and mesowear [4,25-27] methodologies of dietary assessment in fossil ungulates. Due to the complementary nature of these analyses and to the possibility of establishing comparative studies between extant and fossil species, researchers have recently combined some of these techniques to provide more solid inferences of dietary aspects and environment reconstructions for fossils [9,28,29]. It is implied as a second objective to attempt to shed light on the primitive dietary state of the Ruminantia and also to reconstruct the habitat conditions and scenario for the first and broad adaptive radiation of deer in which *Procervulus *was implied.

## Results

Table 1 depicts the results of hypsodonty and teeth wear measurements on Artesilla sample.

**Table 1:** Summary of hypsodonty, microwear and mesowear results.

	Hypsodonty			Microwear			Mesowear					
Species	N	HI [SD]	Hcat	N	S [SD]	P [SD]	N	%H	%L	%S	%R	%B
*Procervulus ginsburgi*	5	0.99 [0.59]	brachydont	13	1153 [475.3]	864 [406.5]	40	92.5	7.5	13.75	75	11.25
-strict browser pattern				2	676 [67.0]	1003 [105.9]	2	100	0	0	100	0
-strict grazer pattern				3	1406 [43.6]	697 [248.6]	2	100	0	33.3	66.6	0

Number of individuals measured (N); hypsodonty index (HI); standard deviation [SD]; hypsodonty category (Hcat); scratches per mm^2 ^(S); pits per mm^2 ^(P); percentage of individuals with high (%H) and low (%L) mesowear relief; percentage of individuals with sharp (%S), rounded (%R) and blunt (%B) cusps. Individuals with strict browser and grazer pattern based on microwear discriminant analysis.

### Analysis of Molar Crown Height

**Figure 1:**
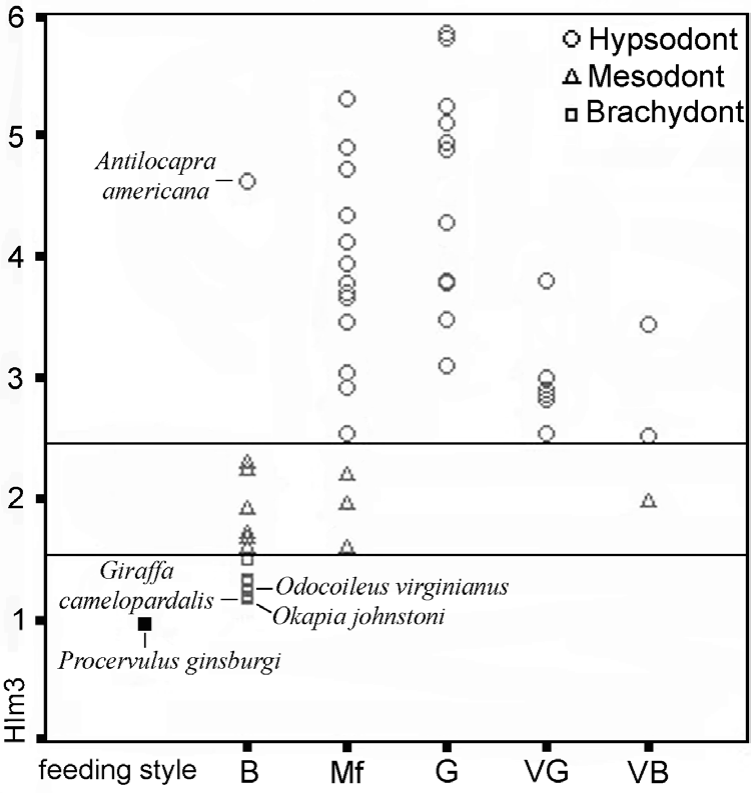
**Feeding style categories plotted against hypsodonty index (HI_m3_) values for the extant taxa and individual fossil species**. Extant browsers (B), mixed feeders (Mf), grazers (G), variable grazers (VG) and variable browsers (VB). Extant data from [1] and [4].

The mean hypsodonty value calculated for *P. ginsburgi *of 0.99 (Fig. 1) falls within the category of brachydont. Given such important presence of low-crowned molars, it should be interpreted as a specialised leaf feeder, well suited for live in closed wood habitats.

Extant ungulates with hypsodonty indices close to 1.0 are *Okapia johnstoni *(1.18), classified as browser in closed habitat, as well as *Giraffa camelopardalis *(1.2) and *Odocoileus virginianus *(1.23) mixed-habitat browsers [1].

Although molar crown height seems to be closely related to dietary preferences, it is not always a reliable predictor of diet in extant ungulates (Fig. 1). All grazing taxa are classified as hypsodonts, and all the grazers *sensu lato *are those with indices of a value greater than 3. Not all the hypsodont species are grazers, however. The decidedly hypsodont dentition of *Antilocapra americana *(4.61), a desert, shrubsteppe and open grasslands dwelling browser [30], has been attributed either to abrasion from grit encroachment on food items [6] or to retention of hypsodonty from ancestors with more abrasive diets [9]. Since the mesowear signal of *A. americana *shows the low relative abrasion of a browser [4], the latter interpretation seems more tenable. The remaining extant browsers are brachydont and mesodont species and there is no ungulate that includes grass in its diet with a lower value than 1.59. None of the extant mixed feeders have brachydont teeth and all the species of this group with a tendency towards grazing (VG) are hypsodonts.

Over evolutionary time scales, molar crown height is in balance with the food-induced wear rate, which results in the well-known relationship between diet and crown height. Although wear rates cannot be calculated for *P. ginsburgi *from the fossil material, it is possible to use wear rates in extant ungulates [31] to bracket the palaeodietary interpretations suggested for the species by tooth wear analyses (Table 2). The mean height of five unworn lower third molars of *P. ginsburgi *is 7.6 mm, and the estimated body mass based on molar measurements [32] is 23.9 kg. Using the regression equation from [31] for estimating longevity from body mass, 0.751 (body mass)^0.29^, 23.9 kg yields an expected life span of 14 years. It appears that *P. ginsburgi *would manage comfortably on a diet similar to that of *Capreolus capreolus*, which tooth-wear rate yields an estimated dental life span of 23 years, much higher than the mass-based estimate of about 14 years. All other wear-based estimates however are significantly lower than the mass-based estimate.

**Table 2:** Estimated size and dental lifespans based on wear rates in selected living ruminants.

	*P. ginsburgi*	*Capreolus*	*Rangifer*	*Gazella*	*Syncerus*	*Bison*
**(a)**	23.92					
**(b)**	13.98					
**(c)**		22.99	8.25	4.66	3.74	2.08
**(d)**		browser	mixed	mixed	mixed	grazer

Body mass; kg (a), mass-based life span; yrs (b), dental life span; yrs (c), conservative feeding style (d). Data from [31].

Those corresponding to the diets of grazing species, 2–4 years, are clearly far too short for a viable population, and even the diet of the mixed feeder *Gazella granti *yields an unrealistically low life span estimate of less than 5 years. Only *Rangifer tarandus*, a mixed feeder close to the browsing end of the spectrum, yields an estimate that may be considered compatible with a viable population, and only so assuming a twinning rate of at least 1.2 (J. Damuth, personal communication based on an unpublished demographic model). Thus, the reindeer could be another ruminant similar to *P. ginsburgi *for which grasses and sedges represent the main part of its food composition (>50%) before seasonal fluctuations and certain survival situations [33].

### Analysis of Microwear Features

**Figure 2:**
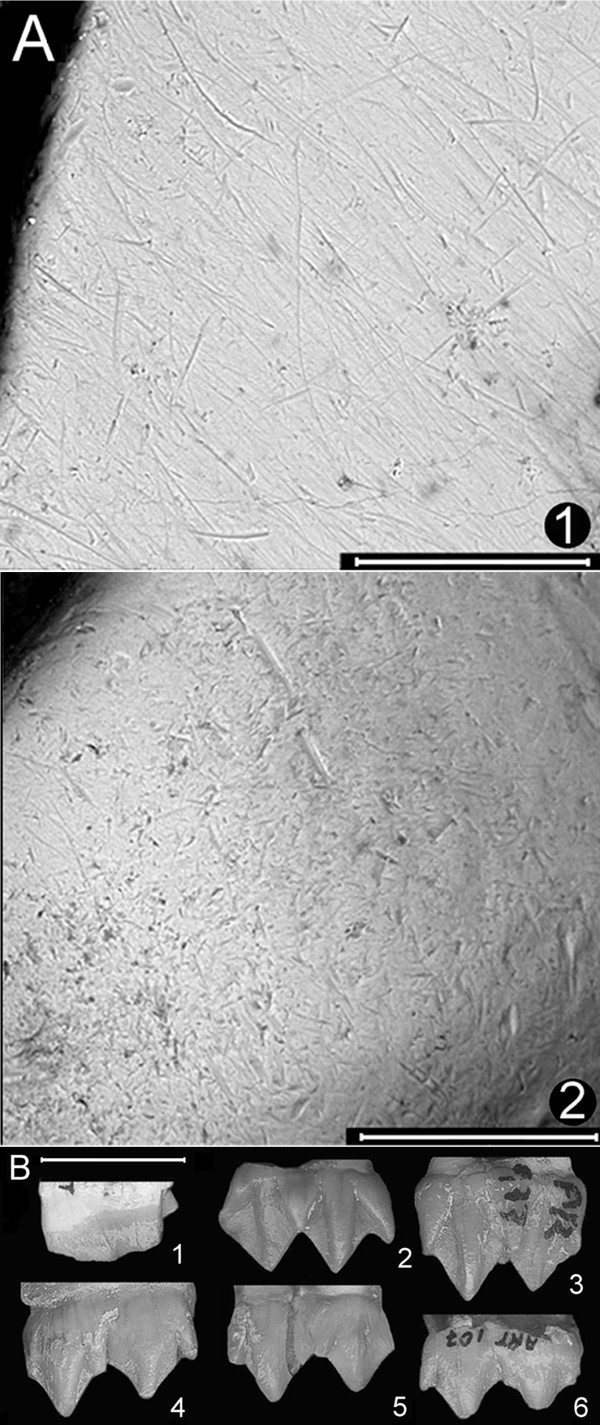
**Photomicrographs of selected fossil tooth enamel and mesowear features**. (A) Photographs at ×500 of two *P. ginsburgi *specimens (scale bar 200 μm). Intensive scratching (1) is probably justifiable as a consequence of the consumption of fresh grasses in a wet season while the intensive pitting (2) suggests a likely intake of browse (fruits, hard seeds) during a dry season. Large pitting seen in both forms of microwear is consistent with the exogenous particle encroachment on food items during the whole year (a pit is defined as large when its maximum diameter exceeds 15 μm). (B) Mesowear features. Low relief and blunt cusps (1;MPZ-2007/7-m_1_; partially broken). High relief and sharp cusps (2;MPZ-6328-M^3 ^and 3;MPZ-6326-M^3^). High relief and rounded cusps (4;MPZ-6353-m_2_, 5;MPZ-6311-M^1 ^and 6;MPZ-2007/6-m_1_). Scale bar 1 cm.

*P. ginsburgi *(Fig. 2A) has enamel surfaces characterised by intensive scratching (1153 scratches/mm^2^) and pitting (864 pits/mm^2^). A clear difference is evident in microwear patterning among the sample (Fig. 2A). The two observed trends are confirmed by statistical analysis (Table 3), with those specimens (23.1%, strict grazers) characterised mainly by an intensive striated enamel surface and those (15.4%, strict browsers) by an enamel surface comprised largely of pitted.

**Table 3:** Summary of microwear discriminant analysis.

Classification rate	82.10%				
	Predicted group				
Original Group	FB	LB	SM	NSM	G
	N(%)	N(%)	N(%)	N(%)	N(%)
**Fruit browser – FB**	2(66.7)	1(33.3)			
**Leaf browser – LB**	2(28.6)	5(71.4)			
**Seasonal mixed feeder – SM**	1(14.3)		6(85.7)		
**Non seasonal mixed feeder – NSM**				4(100)	
**Grazer – G**			1 (14.3)		6(85.7)
**Fossil specimens**		2(15.4)	7(53.8)	1(7.7)	3(23.1)
***P. ginsburgi *average**			1(100)		

Microwear discriminant analysis based on scratch and pit densities.

Intensive scratching usually reflects a large consumption of grasses and grass like plants, since this type of scar results from the abrasion produced by the phytoliths included on cellular walls. Leaves, seeds, and fruits of many ligneous plants also possess phytolith contents, but the frequency is low and unlikely to produce a heavily striated enamel surface [10,11]. Despite the fact that abrasion can be also caused by other abrasive materials, as dust or grit, several authors have identified that the incorporation of these exogenous particles into the diet does not produce differences in the quantity of scratches [6]. Scratch density obtained is much higher than the average for the browsing ungulates (496.3 scratches/mm^2^) and is close to that of grazing ungulates (1231.1 scratches/mm^2^). *Kobus ellipsiprymnus*, *Connochaetes taurinus*, and *Tetracerus quadricornis *(1037, 1069 and 1032 scratches/mm^2^, respectively) are typical grazers and display similar densities than *P. ginsburgi. Ovis canadensis *is another good example of a species inclined to grazing for which the 1235 scratches/mm^2 ^is explained by a 59% of grasses in its diet [34]. Ligneous food preferences are principally responsible for a great abundance of pits [6]. The density recorded for *P. ginsburgi *is much higher than the average for the grazing ungulates (258.2 pits/mm^2^) and even higher to that of browser taxa (495.8 pits/mm^2^). Within the extant browsing, only *Alces alces *has a higher recorded value of density of pits. The mountain grazer *O. canadensis *also displays a great abundance of pits, 1005 pits/mm^2 ^[14]. This sheep occupies rock and steep grounds [35] on which forbs and ligneous plants constitute 19% and 17.5% of its diet, respectively [23,34]. However, this proportion of plants would not be high enough to justify the abundance of pits. From living on dry and dusty habitats, the frequency of pits in the bighorn could be produced if grass blades were picked up from their base, since excessive dust and grit could easily enter the mouth [14]. In the same way as *O. canadensis*, the high pit densities that exceed those of extant browsing species cannot be solely explained by the intake of browse. It also indicates that *P. ginsburgi *ingested a certain amount of inert particles such as dust and grit. Because a large degree of pitting is displayed in most of the specimens, it was likely an encroachment of exogenous material on food items during all year round, while browse was probably enjoyed seasonally or regionally. The relatively pitted signature of the grazer specimens makes persistent grit exposure when these forms incorporated grasses seasonally into their dietary regimes.

**Figure 3:**
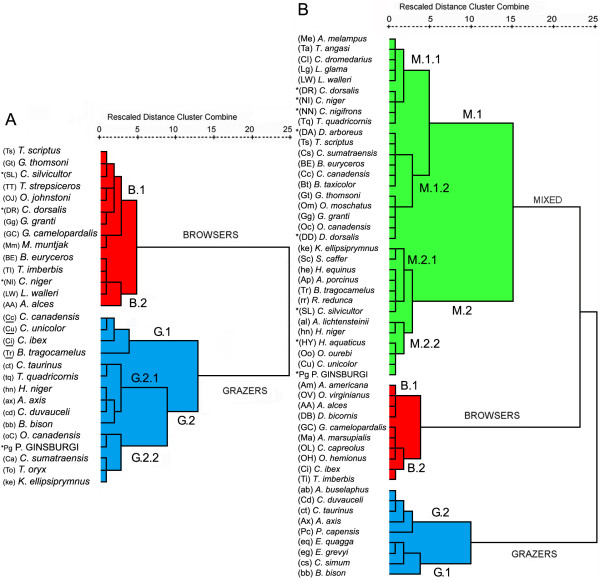
**Hierarchical cluster diagrams**. Microwear hierarchical cluster based on scratch and pit densities features of the reference tooth positions M^2^/m_2 _(A). Mesowear hierarchical cluster based on the tooth positions m_1_–m_3_/M^1^–M^3 ^and percentage high occlusal relief, percentage round cusps and percentage blunt cusps of *Procervulus ginsburgi *with extant species (B). Shared symbols: UPPER CASE = LEAF-BROWSER, UPPER CASE WHIT ASTERISK = FRUIT-BROWSER, lower case = grazer, Mixed case with asterisk = Fossil species. Exclusive microwear symbols: Non-underlined Mixed case (Capital first) = Seasonal Mixed feeder, Underlined Mixed case (Capital first) = Non-seasonal Mixed feeder, mixed Case (lower First) = mountain Grazer. Exclusive mesowear symbols: Mixed Case (Capital first) = Mixed Feeder. Extant wear data from [4] and [14].

We explore the similarities among the microwear pattern of *P. ginsburgi *and 28 current ungulates by means of hierarchical cluster analysis (Fig. 3A). The taxa are distributed into two main clusters separating clearly those with a preference to browse fruits and leafs (cluster Browsers) from those of graze (cluster Grazers). The fossil deer appears grouped in this second main cluster close to *O. canadensis*, *C. sumatraensis*, *T. oryx *and *K. ellipsiprymnus *(subcluster G.2.2). A probable seasonal preference for graze is appreciated in this group of taxa. The species included in this subcluster, with the exception of *K. ellipsiprymnus*, show two distinct forms of microwear; one typical of browsing, and one of grazing (Fig. 2A), because some of the individuals browsed right before death while others grazed [13]. This fact is traditionally considered as a seasonality signal.

We use discriminant analysis to force the classification of *P. ginsburgi *into an established feeding style. In order to avoid a decrease in the discriminant consistency, the mountain grazer feeding style was not considered in the analysis because it is only formed by *O. canadensis*. Consequently, this species was included as a seasonal mixed feeder [6].

The analysis was developed using as criterion variables density of scars (Table 3). A very high discrimination is given among the 28 extant ungulates with a classification rate of 82.1%. It must be noted that most misclassifications were realized from the leaf-browser toward the fruit-browser style, and in the reciprocal direction. *Cephalophus niger *stands out in terms of having scratch densities within the leaf browsing scratch range. The apparent consumption of fruits and seeds with relatively soft coverings [6], compared to the other fruit browsers, can be the explanation for this microwear pattern. *Muntiacus muntjak *mixed feeder was placed in the category of fruit browsers. However, there are individual differences which may have to do with the relative hardness of fruit coverings and/or seed coats or with the relative amounts of these items consumed. These differences result in a variable number of scratches [6]. Importantly, no species were classed as a grazer while being a browser, and vice versa.

**Figure 4:**
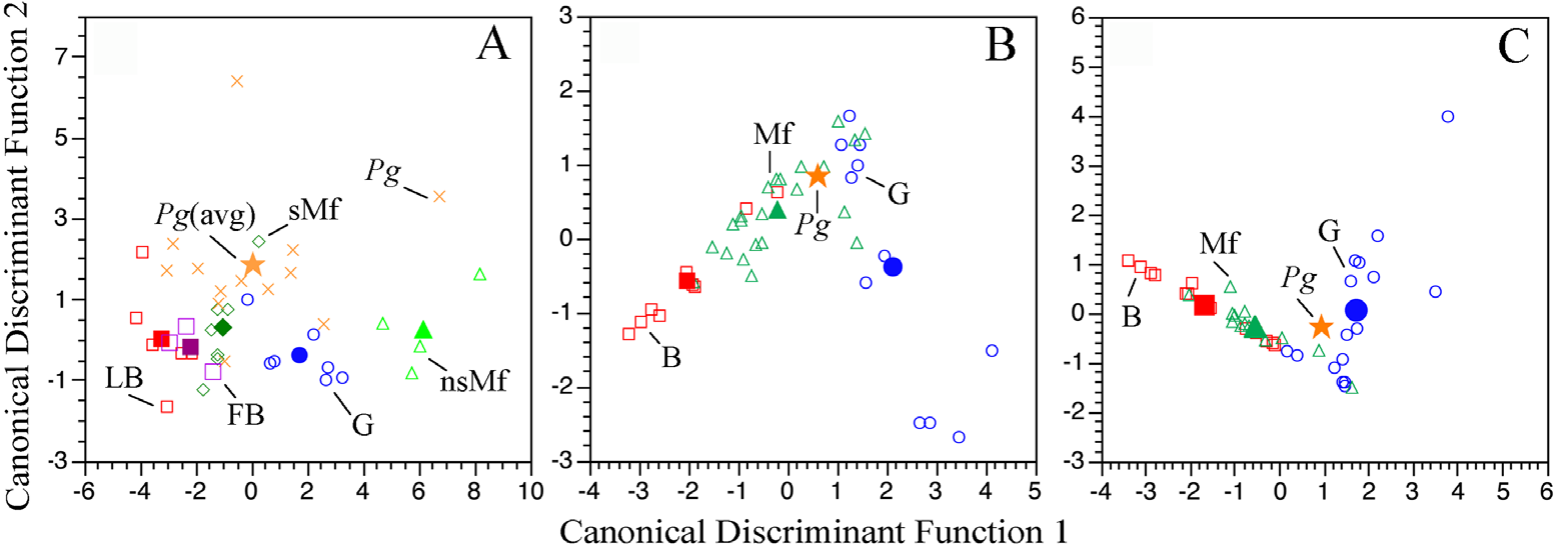
**Distribution of ungulate species in the morphospace defined by the microwear (A) and mesowear conservative (B) and radical (C) discriminant analyses models**. Share symbols: extant fruit and leaf-browsers (FB, LB; *open squares*), grazers (G; *open circles*), *P. ginsburgi *(Pg; *closed star*). Exclusive microwear symbols: extant non-seasonal (nsMf; *open triangles*) and seasonal mixed feeders (sMf; *open rhombus*), *P. ginsburgi *specimens (*cross*). Exclusive mesowear symbols: extant mixed feeders (Mf; *open triangles*). *Closed *symbols representing centroids. Extant data from [4] and [14].

Most of the specimens, as well as the average of *P. ginsburgi*, were classified as seasonal mixed feeders (Table 3). These specimens fall between the browser and grazer domains, and some others fall into the grazers and others fall into the browsers (either fruit or leaf dominated, Fig. 4A). By displaying affinities from these different trophic categories, the wear produced by the last items consumed prior to death classifies *P. ginsburgi *into the seasonal mixed category (Table 3, Fig. 4A).

### Analysis of Mesowear Features

**Figure 5:**
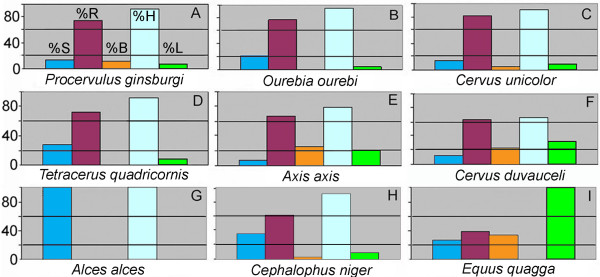
**Histograms of mesowear variables scored**. Percentage of individuals with sharp (%S), round (%R) and blunt (%B) cusps; percentage of individuals with high (%H) and low (%L) mesowear relief. Histogram of *P. ginsburgi *(A) is based on the values given in Table 1. Comparative histograms based on published data from [4]. Mixed feeder species (B-F); leaf (G) and fruit (H) browser species; grazer species (I).

The mesowear signal seen in *P. ginsburgi *is characterised by the predominance of round cusps (75%) and high relief (92.5%) and also by significant proportions of sharp (13.75%) and blunt (11.25%) cusps (Table 1, Fig. 2B). This pattern responds to that found in species with some abrasive diets and is clearly different to both the strict browser and strict grazer taxa. The percentages of cusp shape and occlusal relief are close to those of the Indian monsoon forest *Cervus duvauceli*, *Axis axis*, *Cervus unicolor *and *Tetracerus quadricornis *and to the African *Ourebia ourebi *conservative mixed feeders [4]. All these species show a highly abrasion-dominated wear, but only *C. duvauceli*, and *A. axis *show a grazerlike profile with over 20% of blunt cusps (Fig. 5). In fact, both species together with *T. quadricornis *and *O. ourebi *are indeed treated as grazers in the "radical" classification [4]. Some recent studies even report that the *O. ourebi *is a pure grazer [36]. In fact, particularly interesting is its behaviour, since it is considered the most primitive of the extant grazing ruminants concerning the feeding style [37]. It grazes at least eleven different herbs during the wet season when fresh grass is readily available, and it browses the foliage from seven different trees when drought occurs and fresh grass is less common [37].

The similarities among the mesowear pattern of *P. ginsburgi *and a set of 51 well-known extant species [4] were also obtained by means of hierarchical cluster analysis. The taxa are distributed into three main clusters separating clearly those with mixed dietary trends (cluster Mixed) from those with preference to browse (cluster Browsers) and to graze (cluster Grazers). *P. ginsburgi *appears clearly separated from the browser taxa. The fossil deer is thus grouped with *C. unicolor*, and *O. ourebi *mixed feeders and with *Hyaemoschus aquaticus *fruit browser. It also appears close to *A. lichtenstanii *and *H. niger *grazers (in the subcluster M.2.2, Fig. 3B). Removing the problematic fruit-browser class ("mabra" group, see methods section), did not affect the relative placement of the remaining species, and even *P. ginsburgi *fell in homologous clusters between the different data sets.

Discriminant analyses were performed to quantify the resolution of mesowear analysis applied to *P. ginsburgi *with respect to the four conventional dietary classes of fruit and leaf browser, grazer, and mixed feeder. The combination of rounded, blunt cusps and high relief and two dietary classifications (conservative and radical) alternately as a grouping variable was used in this study (Table 4).

**Table 4:** Summary of mesowear discriminant analyses.

	A				B			C		
Classification rate	56.90%				76.70%			74.40%		
	Predicted group				Predicted group			Predicted group		
Original Group	FB	LB	M	G	LB	M	G	LB	M	G
	N(%)	N(%)	N(%)	N(%)	N(%)	N(%)	N(%)	N(%)	N(%)	N(%)
**Fruit browser – FB**	3(42.9)		4(57.1)							
**Leaf browser – LB**		7(77.8)	2(22.2)		7(77.8)	2(22.2)		8(61.5)	5(38.5)	
**Mixed feeder – M**	7(29.2)	3(12.5)	12(50.0)	2(8.3)	3(13.0)	17(73.9)	3(13.0)	2(15.4)	9(69.2)	2(15.4)
**Grazer – G**	4(36.4)			7(63.6)		2(18.2)	9(81.8)		2(11.8)	15(88.2)
***P. ginsburgi *average**	1(100)					1(100)				1(100)

Mesowear discriminant analyses for the conservative with "mabra" (A) and for both the conservative (B) and the radical (C) dietary classifications without "mabra".

For the full dataset of 51 extant ungulates, the mean percentage correctly classified was only 56.9% for conservative (Table 4A).

This discrimination is not high enough to be sure of the trophic assignments and could be therefore partially or completely masked by non typical browsers.

Among the 22 species wrongly classified, 4 species involved in frugivory (*C. dorsalis*, *C niger*, *D. arboreus *and *D. dorsalis*) were wrongly assigned to the mixed feeder category. Moreover, 7 mixed feeders and 4 grazers fell within the fruit browser category, respectively. *P. ginsburgi *was also assigned to the fruit browser class. Although fruit browsers and *P. ginsburgi *have similar rounded apices, it should be emphasized that the difference is otherwise highly important regarding the percentage of blunt cusps (Fig. 5). Species that are well known to be highly frugivorous have a small percentage of blunt cusps, probably because of tip-crushing wear due to frugivory [4]. The rounding of their cusps is also probably due to this. Blunt cusps are only seen in *Cephalophus*, less than 5% in most of cases, while absent in *Heterohyrax *and *Dendrohyrax*. Therefore, an important degree of dental abrasion is displayed by *P. ginsburgi*, which is probably justifiably interpreted as proportion of grass in the food. In line with this case, the dental wear analysis of extinct brachydont *Rangifer *with extremely blunt teeth, clearly shares strong affinities with extant mixed feeders [38].

Because of this abrasion-dominated mesowear signal, as discussed above, *P. ginsburgi *could be one of these several mixed feeder species misclassified as frugivorous.

When the problematic "mabra" group [4] was weeded out of the data, set the percentage of correctly classified notably increased for both the conservative (76.7% correct) and radical (74.4% correct) dietary classifications (Table 4B,C). Then, the most probable dietary assignment for *P. ginsburgi *based on the long term dietary effect corresponds either to a mixed feeder according to the conservative classification, or to a grazer according to the radical (Table 4B,C, Fig. 4).

## Discussion

Data and arguments from two independent methodologies based on dental wear failed to conclude that *Procervulus ginsburgi *was a browser, as can be expected by the considerable brachydont cheek teeth. Conversely, the Artesilla extinct species is firmly placed close to mixed feeder extant species displaying that brachydonty does not necessarily imply exclusive browsing in deer.

Today, the use of grass and browse by herbivores depends, to a large extent, on competition between species of herbivores, food availability and avoidance of predation [5]. Before the advent of species whose teeth present morphologies that suggest adaptations for grass-eating, especially of bovids, *P. ginsburgi *could have had a greater relative grazing capacity and was able to digest a higher grassy diet than was previously expected for a brachydont species today. A wear rate at the lower end of the mixed feeder range would be supported assuming a twinning rate of at least 1.2 to keep a viable population. It appears that *P. ginsburgi *could have managed comfortably on a seasonal mixed feeding style grazing probably fresh grasses in the wet season and browsing various foliages, seeds and fruits during the dry season.

This relative inferred grazing capacity, and therefore grass availability, is also consistent with the vegetation data. Little information exists about plant remains from the Calatayud-Daroca basin, but recent data [39] confirm the presence and significance of graminoids in the nearby Lower Miocene Rubielos de Mora basin (Teruel, Spain). The evidence of *Myrica *and *Cornus *genera and some *Lauraceae *family shrubs, also found in this basin, could easily have constituted part of the ligneous material consumed by *P. ginsburgi*.

The palaeoenvironmental reconstruction of Artesilla would be quite imprecise, raising doubts about how open the habitat was or how widespread were grasses. However, high pitting produced by the last items consumed prior to death of *P. ginsburgi*, cannot be only explained by browse, and may reflect more arid and open habitats than were supposed in previous reconstructions, where the environmental conditions would facilitate the deposit and the adhesion to food items of grit and dust. Since some seasonal food differences have also been detected, it may have occupied different areas during the seasonal cycle. These interpretations lead us to conclude that during the latest Early Miocene (Zone C, MN4), *P. ginsburgi *would arise at a time of increasing aridity, openness and seasonality in the Calatayud-Daroca basin.

**Figure 6:**
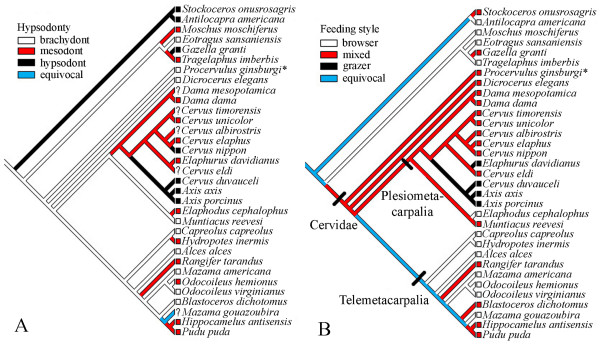
**Reconstructing evolutionary history of hypsodonty (A) and feeding style (B) in Cervidae**. Characters are traced onto a tree topology based on a recent molecular phylogeny [55] using MacClade 4.0.6 [56]. Two outgroups are considered for tracing displays of ancestral character states, *A. americana *and a clade formed by *G. granti *and *T. imberbis *bovids with *M. moschiferus*, following the mentioned molecular analysis. Data of extant taxa from [1], [5] and [9]. *P. ginsburgi *and *Dicrocerus elegans *are included in the cladogram as stem representatives of extant cervids. Also included were *Stockoceros onusrosagris *antilocaprid [9], closely related to *A. americana*, and *Eotragus sansaniensis *bovid [11] to obtain more reliable ancestral characters. The ordered type is assigned for reconstructing the transitions of feeding style from assumptions reported in [5], while the irreversible type is assumed to be the hypsodonty evolution [21]. Dental wear data of extinct species from [9] and [13].

Data presented here for *P. ginsburgi *reveal that grazing habits in the Cervidae were acquired prior to the development of hypsodonty (Fig. 6A). High-crowned cheek teeth do not usually appear to be decreased in evolution once it has evolved [21]. However, regarding feeding, the mixed feeder state is a flexible evolutionary state that acts as a link between browser and grazer states [5]. Thus, the three established feeding styles were acquired independently in various lineages of both telemetacarpal and plesiometacarpal deer having in some cases, no relationship with the evolution of hypsodonty (Fig. 6B). Assuming the ancestral browser state for cervids to be true [11,15], this would result in the equivocal character tracing on certain branches especially in the node for plesiometacarpal deer. If *Dicrocerus elegans *mixed feeder [13] is included in the cladogram, the equivocal tracing is extended to the node for Cervidae. This ambiguity is resolved when an ancestral mixed feeding state is assumed to be present in *P. ginsburgi*. These data corroborate previous studies [13] that suggested that mixed feeding was not only a transitional state and link between browsing and grazing [5], but also, and more important, the initial state from which both were evolved. Earliest cervids could have been physiologically qualified to browse and graze, but the morphological expression of this facultative mixed condition would have depended on environmental conditions. Therefore, it could be expected that a variable spectrum of feeding strategies, from strict browsing to grass-dominated mixed feeding, will be found among Early Miocene cervids without any hypsodonty increase would have taken place.

Was this facultative mixed state a first advantage exclusive of Cervidae or could it also have been the starting point of other ruminant lineages? The mixed feeder strategy newly obtained for *P. ginsburgi *contributes to tip the balance in favour of a mixed condition as the primitive dietary state of the Ruminantia. In fact, the evolutionary scenario built to Cervidae seems to be about the same that the already found for other ruminant clades.

Regarding low-crowned molar groups, and therefore traditionally characterised as browsers, gross craniodental analysis and wear methodologies gathered from some extinct Giraffidae and Dromomerycidae have revealed the appearance of grazing adaptations early on in their evolution [7,10,14]. Interestingly, if a wider range of dietary habits than was previously expected for their brachydonty is early represented in the families, an ancestral mixed condition could have also been feasible.

With respect to hypsodont clades, the earlier and more primitive merycodonts which were apparently qualified to graze from their first occurrences, both browsed and grazed seasonally and regionally, despite having these high tooth crowns. Also, the first antilocaprines were mixed feeders, while the latter apparently consumed grass on a more frequent basis and shifted to browsing dietary regimes only in the early Pleistocene [40].

As far as we know, the only other data regarding early information available, concern the archaic bovid *Eotragus sansaniensis *from the Middle Miocene (MN6) locality of Sansan (France). Results from our study may shed light to propose a particular evolutionary scenario and thus explain the apparent contradiction that supposes the browsing diet [11] of *E. sansaniensis*. If increased molar crown height is recognized as an adaptation for grass eating, it should be assumed that this bovid, displaying a higher degree of hypsodonty (although still brachydont) than *P. ginsburgi *mixed feeder, would evolve from an ancestor that enjoyed more grasses in the past. In fact, the oldest known horned bovid of Africa, *Namacerus gariepensis *(ca 17.5 Ma), could already have incorporated grass in its diet having a precocious grade of hypsodonty [41]. In Sansan locality, *E. sansaniensis *is the only bovid and it coexisted with another five ruminant taxa, of which cervoids are clearly predominant. Its molar height should suggest at least a mixed feeding, and however, in the faunal assemblage of Sansan, the large *Dicrocerus elegans *occupied this niche. The high abundance of *D. elegans*, an exclusive case in the Miocene ruminant fauna, would support a gregarious behaviour coherent with the fact of being the only extinct deer with antlered females [42], and only comparable with the extant *Rangifer*. It is perhaps not surprising to think that in a flourishing time of cervoid groups, they were prone to inhabit a broad range of feeding niches. Therefore, it was not easy for *Eotragus*, the first immigrant bovid in Europe, to replace deer if they were perfectly established in the mixed niche, as the scarce available data suggest. Since the Sansan diversity of ruminants was large, dietary differentiation may have been necessary for the survival of *E. sansaniensis*. Thus, it could be compatible with our assumptions that *E. sansaniensis *would have occupied a browser regime due to strong competition, although being physiologically and morphologically adapted to feed on mixed diet.

Ruminant ancestor, and even basal clades as tragulids, consumed a variety of different vegetations [6], they may also have ingested animal matter, despite the brachydont nature of their cheek teeth. Similarly, early pecorans could have preserved a certain degree of this opportunism through a mixed feeding strategy. Higher ruminants could have been physiologically qualified to both browse and graze, i.e. a facultative mixed state, from their first appearance. Therefore, vegetal resources accessibility, climatic conditions and competition between species could have been the factors in controlling the expression of different feeding styles. Browse-eating would be the most efficient specialization for obtaining required nutrients when a continued predominance of high nutritional forage is prevailing, despite the possible existence of grasses. If seasonality and aridity restrict the nourishing vegetal resources, ruminants would be forced to display mixed feeder strategies to extract nutrients from a wide variety of vegetation. Early grass-dominated mixed feeders would thus have grazed only for a short season each year, but this might have been crucial for their survival. Exclusive grazing habits would arise at a time of increasing seasonal dryness and development of more open habitats, as an adaptation to increase harvesting efficiency and reduce food search (since grasses typically grow in continuous dispersion). In fact, climates became drier and more seasonal in the middle Miocene when the Antarctic ice sheet was near-permanent [43]. Grazing species, therefore, could invest less time by making decisions on how to maximize nutrient intake than did browsers, and could therefore gain time for other essential activities, such as reproducing and avoiding predators [44]. Finally, browse specialization would become possible just after the spreading of open habitats if competition between ruminant herbivores was increased.

## Conclusion

Despite the traditional characterization of first deer as specialised leaf-eating, our analysis of molar crown height and dental wear strongly supports that *Procervulus ginsburgi*, while having low-crowned molars, exhibited a strategy typical of seasonal mixed feeders. Thus, it seems more likely that Cervidae were originally leaf-grass mixed feeders. Important dietary heterogeneity among other ruminant clades suggests that a facultative mixed state may in fact have been the primitive dietary state of the Ruminantia, which would have been morphologically expressed only under specific environmental factors. However, more palaeodietary investigations on first records of early taxa from different localities are nevertheless needed to validate the presumptions proposed here. In the light of these data, future palaeoecological studies should take into account that some terms, such as "low-crowned molars", "leaf-eating species" and "forest environments", are not strictly associated, at least not for the Cervidae.

## Methods

### Location and Data Collection

The Aragonian deposits of Artesilla (Lower Miocene) near Villafeliche (province of Zaragoza, North Central Spain), have been the object of several palaeontological excavations during the ninetieth. Magnetostratigraphic [45] and biostratigraphic [46] analyses in the Aragonian Type section have dated Artesilla at 16.51 Ma and placed it to the local Zone C and Mein'zone MN4.

Very well preserved *P. ginsburgi *upper (*n *= 14) and lower (*n *= 26) molar teeth belonging to a minimum of 21 individuals from the deposit of Artesilla were available for study. The specimens are housed at the Museo Paleontológico de la Universidad de Zaragoza (Zaragoza, Spain).

### Associated Fauna

The faunal assemblage is varied and extensive, with at least 38 vertebrate taxa of which 20 are large mammals. Despite the presence of *Megacricetodon collongensis*, Zone C cricetid indicator, Van der Meulen and Daams [47] considered the association more similar to Zone B due to the scarcity of the forest dweller *Ligerimys*. Artesilla site also represents the oldest record of possible African origin immigrants as creodonts (*Hyainailouros*), felids (*Afrosmilus*) and Proboscideans (*Prodeinotherium *and *Gomphotherium*). The fossil bovids also constitute part of these immigrants in zone C but they are not present in Artesilla. Ruminants are dominated by the cervoid taxa *Procervulus *and *Ampelomeryx*. Due to the predominance of brachydont species as well as the presence of deinotherids and tragulids, the large mammal association was related with a closed and humid palaeoenvironment [48].

### Dietary Assessment Methodologies

Several techniques developed in recent years have focussed on reconstructing dietary preferences in ungulates. Craniodental morphological and morphometric analyses have divided ungulates into the known categories [2,49,50]. However, they cannot be applied because they require the complete preservation of specific pieces which are not available in our sample. Stable isotope techniques have also tested a connection between the isotopic composition of tissues records and diet and have provided important dietary insights [51,52]. Since the study is based on the carbon isotopic distinction between C3 and C4 photosynthesis and due to C4 monocots is not detected in the Mediterranean region [51] where Artesilla material was collected, it is not possible to document dietary preferences from stable carbon isotope data. Thus, only the following procedures can be used in the research for interpreting the dietary regime in *P. ginsburgi *and reconstructing the environment of Artesilla.

#### Dental Crown Height

To test the relationship between hypsodonty (i.e. the increased molar crown height) and feeding style, the lower third unworn molar teeth (*n *= 5) were measured using an index (HI_m3_) defined as the unworn m_3 _height divided by m_3 _width (1,3), and classified "brachydont ungulates" as a HI_m3 _< 1.5, "mesodont ungulates" as a 1.5 < HI_m3 _< 2.5 and "hypsodont ungulates" as a HI_m3 _> 2.5.

#### Dental Microwear

The method applied for this study was performed following a procedure previously developed [11] and widely used by other authors.

Microscopic scar observation was made by means of an Environmental Scanning Electron Microscope FEI Quanta 200. Images were taken systematically at a magnification of ×500 in a 0.20 mm^2 ^delimited area and were treated with Microware 4.02 [53]. Microwear dimensions were directly categorized on the basis of the ratio length/width (pits: ratio = 4, scratches: 4 < ratio < 100) by this software.

The study examined the anterior lingual blade of the paracone on the M^2 ^(*n *= 4) and the posterior buccal blade of the protoconid on the m_2 _(*n *= 9) permanent.

Dental microwear comparisons were made using a dataset [14] partitioned into the leaf browsing (*n *= 7), fruit browsing (*n *= 3), grazing (*n *= 7), mountain grazer (*n *= 1), seasonal (*n *= 6) and non-seasonal mixed feeder (*n *= 4) categories. Note that the "non-seasonal mixed feeder" term is synonym of "meal by meal mixed feeder" from [6].

Number of scratches and pits, variables commonly adopted for analysis, were transformed into a density score [23] in order to simplify the comparison of *P. ginsburgi *with other extant ungulates. Quantitative analysis was performed using the dataset of 28 well-known extant ungulates compiled by [14] and sharing the same protocol concerning high magnification (×500) and SEM.

#### Dental Mesowear

Occlusal relief (characterized as either high or low) and the cusp shape (characterized as either sharp, rounded, or blunt) of the apex of the paracone and metacone of the M^1^–M^3^(*n *= 14) and the metaconid and entoconid of the m_1_–m_3 _(*n *= 26) were examined by the naked eye (and occasionally using a stereomicroscope Olympus SZ11) and qualitatively scored. A comparative extant database with known diets was followed as a reference [4] from which small samples represented by less than 15 individuals were removed in order to obtain a range in which distributions that look different become significantly different statistically. The mesowear pattern stabilizes after about 20 or 30 individuals, and usually gives a reasonable approximation after about 10 [4]. The dental mesowear of fossil species was then compared to a set of 51 well-known extant ungulates composed of conservative fruit (*n *= 7), and leaf browsing (*n *= 9), conservative grazing (*n *= 11) and conservative mixed feeder species (*n *= 24). The duikers and hyraxes constitute part of the fruit-eaters as a special case ("mabra", for "minute abraded brachydont") showing quite unclear dental wear. Note also that some doubtful cases are treated as intermediate (mixed feeder) in the conservative classification and as extreme (browser or grazer) in the radical classification and could be considered variable browsers and variable grazers, respectively. As recommended by [31], we used all available upper molars except for those in very early and very late wear. Because the mesowear method relies on the wear process itself, it is least reliable for the most brachydont species [31], and the results for *Procervulus *must therefore be treated with particular caution.

### Statistical Analyses

Multivariate analyses for ascertaining the most probable feeding style in *P. ginsburgi *were performed using SPSS 11.5 [54]. Hierarchical cluster analysis was applied as an explorative technique for identifying herbivorous species groups and emphasizing any inherent structure, based on similarities in wear pattern. The analysis was realized using the Euclidean distance and Ward's method. Discriminant analysis was also employed to evaluate the ability of the dental variables set with the purpose of distinguishing between the known feeding styles of extant species and also to classify the *P. ginsburgi *cases according to the model derived.

It was found that cluster diagrams with an index of hypsodonty showed no difference with that obtained when this variable was excluded. It has also been proved that discriminant models that include bovids as a part of an extant data set are prone to classify the fossil taxa as browsers when hypsodonty is included [7]. For these reasons and through being considered an independent methodology in research, hypsodonty was reasonably excluded from the analyses based on dental wear.

## Abbreviations

Mabra: minute abraded brachydont; MN: mammal Neogene zone; MA: million years; KG: kilogramme; YRS: years; CM: centimetre; MM^2^: square millimetre

## Authors' contributions

DDM, BA and JM planned the study and designed the research. All authors performed the research. DDM, MF and BA contributed to the analysis of the data and to the writing of the paper. All authors read and approved the final manuscript.
